# Supplementary material for: Comparative Study of Green and Traditional Routes for Cellulose Extraction from a Sugarcane By-Product
Source: Polymers (Basel). 2023 Mar 1;15(5):1251. doi: 10.3390/polym15051251 (PMC10007196; doi:10.3390/polym15051251)
Supplement: Supplementary file 1 [file polymers-15-01251-s001.zip › polymers-2230603-supplementary.pdf]

Comparative Study of Green and Traditional Routes for Cellulose Extraction from a Sugarcane by-product

Francisca Casanova[1,a], Ricardo Freixo[1,a], Carla F. Pereira[1,*], Alessandra B. Ribeiro[1], Eduardo M. Costa[1], Manuela E. Pintado[1] and Óscar L. Ramos[1]

A contribution from

[1]Universidade Católica Portuguesa, CBQF - Centro de Biotecnologia e Química Fina – Laboratório Associado, Escola Superior de Biotecnologia, Rua Diogo Botelho 1327, 4169-005 Porto, Portugal.

[a] These authors contributed equally to this work as first authors.

Electronic Supporting Information

All correspondence should be addressed to:

Carla F. Pereira: cpfpereira@ucp.pt

Table S1 – FT-IR vibration bands assignments for all products obtained from different cellulose extraction methods applied to sugarcane bagasse.

Wavenumber (cm^{-1})	Assignments	Samples
830	C–H stretching in lignin aromatic rings [1]	SCB, AuH, ELa1:2, AcH<br>SCB>315, AuH_170, AuH_180, AuH_190, EtOH
900	C-H rocking in cellulose (β-glycosidic linkages) [2]	All
1030	C–O stretching in cellulose [1]	All
1060	C-O-C ring stretching in cellulose [3]	CC, AuH<br>AuH_170, AuH_180, AuH_190, Au_EtOH
1110		CC, AuH, ELa1:2<br>AuH_170, AuH_180, AuH_190, Au_EtOH
1160		All
1250	C-O stretching of guaiacyl ring in lignin [4]	SCB, ELa1:2, AcH<br>SCB>315, EtOH
1370	C-H and C–O bending in aromatic rings of polysaccharides [5]	All
1430	CH_2 bending associated with crystalline cellulose [1]	All
1500	C=C stretching of aromatic ring in lignin [6]	All
1600-1640	O–H bending due to adsorbed water in cellulose [3]	All
1730	C=O stretching vibration of the acetyl and ester linkages in lignin and hemicellulose [2]	SCB, ELa1:2, AcH<br>SCB>315, EtOH
2850	CH_2 bending associated with hemicellulose [7]	SCB, ELa1:2, AcH, AlkH<br>SCB>315
2900	C-H stretching in cellulose [6]	All
3200-3400	O-H stretching of intramolecular hydrogen bonds in cellulose [8]	All

Legend: CC: commercial cellulose; SCB: untreated sugarcane bagasse; AuH: fraction extracted by autohydrolysis; ELa1:2: fraction extracted by DES with choline chloride and lactic acid (ratio 1:2); AlkH: fraction extracted by alkaline hydrolysis; AcH: fraction extracted by acid hydrolysis; SCB>315: untreated sugarcane bagasse fraction superior to 315 μm; AuH_EtOH: fraction extracted by autohydrolysis followed by organosolv with ethanol; EtOH: fraction extracted by organosolv with ethanol; AuH_190: fraction extracted by autohydrolysis at 190 °C; AuH_180: fraction extracted by autohydrolysis at 180 °C; AuH_170: fraction extracted by autohydrolysis at 170 °C.

References

1. Md Salim, R.; Asik, J.; Sarjadi, M.S. Chemical Functional Groups of Extractives, Cellulose and Lignin Extracted from Native Leucaena Leucocephala Bark. *Wood Sci. Technol.* **2021**, *55*, 295–313, doi:10.1007/S00226-020-01258-2/TABLES/1.

2. Li, R.; Fei, J.; Cai, Y.; Li, Y.; Feng, J.; Yao, J. Cellulose Whiskers Extracted from Mulberry: A Novel Biomass Production. *Carbohydr. Polym.* **2009**, *76*, 94–99, doi:10.1016/J.CARBPOL.2008.09.034.

3. Kumar, A.; Negi, Y.S.; Choudhary, V.; Bhardwaj, N.K. Characterization of Cellulose Nanocrystals Produced by Acid-Hydrolysis from Sugarcane Bagasse as Agro-Waste. *J. Mater. Phys. Chem.* **2014**, *2*, 1–8, doi:10.12691/jmpc-2-1-1.

4. Chourasia, V.R.; Pandey, A.; Pant, K.K.; Henry, R.J. Improving Enzymatic Digestibility of Sugarcane Bagasse from Different Varieties of Sugarcane Using Deep Eutectic Solvent Pretreatment. *Bioresour. Technol.* **2021**, *337*, 125480, doi:10.1016/J.BIORTECH.2021.125480.

5. Wulandari, W.T.; Rochliadi, A.; Arcana, I.M. Nanocellulose Prepared by Acid Hydrolysis of Isolated Cellulose from Sugarcane Bagasse. *IOP Conf. Ser. Mater. Sci. Eng.* **2016**, *107*, doi:10.1088/1757-899X/107/1/012045.

6. Saelee, K.; Yingkamhaeng, N.; Nimchua, T.; Sukyai, P. Extraction and Characterization of Cellulose from Sugarcane Bagasse by Using Environmental Friendly Method. In Proceedings of the The 26 th Annual Meeting of the Thai Society for Biotechnology and International Conference ; 2014.

7. Indran, S.; Edwin Raj, R.; Sreenivasan, V.S. Characterization of New Natural Cellulosic Fiber from Cissus Quadrangularis Root. *Carbohydr. Polym.* **2014**, *110*, 423–429, doi:10.1016/J.CARBPOL.2014.04.051.

8. Saravanakumar, S.S.; Kumaravel, A.; Nagarajan, T.; Sudhakar, P.; Baskaran, R. Characterization of a Novel Natural Cellulosic Fiber from Prosopis Juliflora Bark. *Carbohydr. Polym.* **2013**, *92*, 1928–1933, doi:10.1016/J.CARBPOL.2012.11.064.
